# Neglect in Human Communication: Quantifying the Cost of Cell-Phone Interruptions in Face to Face Dialogs

**DOI:** 10.1371/journal.pone.0125772

**Published:** 2015-06-03

**Authors:** Matías Lopez-Rosenfeld, Cecilia I. Calero, Diego Fernandez Slezak, Gerry Garbulsky, Mariano Bergman, Marcos Trevisan, Mariano Sigman

**Affiliations:** 1 Laboratorio de Inteligencia Artificial Aplicada, Departamento de Computación, Facultad de Ciencias Exactas y Naturales, Universidad de Buenos Aires, Pabellón 1, Ciudad Universitaria, 1428 Buenos Aires, Argentina; 2 Laboratorio de Neurociencia, Universidad Torcuato Di Tella, Av. Figueroa Alcorta 7350, (C1428BCW) Ciudad de Buenos Aires, Argentina; 3 El Mundo de las Ideas, Buenos Aires, Argentina; 4 Departamento de Física, Facultad de Ciencias Exactas y Naturales, Universidad de Buenos Aires, Buenos Aires, Argentina; University of Rijeka, CROATIA

## Abstract

There is a prevailing belief that interruptions using cellular phones during face to face interactions may affect severely how people relate and perceive each other. We set out to determine this cost quantitatively through an experiment performed in dyads, in a large audience in a TEDx event. One of the two participants (the speaker) narrates a story vividly. The listener is asked to deliberately ignore the speaker during part of the story (for instance, attending to their cell-phone). The speaker is not aware of this treatment. We show that total amount of attention is the major factor driving subjective beliefs about the story and the conversational partner. The effects are mostly independent on how attention is distributed in time. All social parameters of human communication are affected by attention time with a sole exception: the perceived emotion of the story. Interruptions during day-to-day communication between peers are extremely frequent. Our data should provide a note of caution, by indicating that they have a major effect on the perception people have about what they say (whether it is interesting or not . . .) and about the virtues of the people around them.

## Introduction

Cellular phones are ubiquitous, with more than 300 million users in the United States and more than 6.800 millions world-wide [1]. This pervasive device opens a window to understand multiple aspects of human society. Several studies have analyzed cell-phone calls as large-scale social networks, describing communication patterns and network structure [2, 3, 4, 5, 6]. Communication data has been studied to understand human mobility patterns [7, 8, 9]. These data also offers an indirect measure for social interactions—showing non-Poissonian bimodal interevent distributions [10, 11, 12]—and where relationships between people may be infered from reciprocal calls patterns [13, 14, 15].

These studies focus principally on cell-phones as a tool for quantitative sociological research to understand different aspects of human behavior. Instead, we investigate the impact that cell-phone distraction may have in human relations. Cell-phone use impairs attention even to gazed elements of the visual scene [16], which has been recognized as a major risk factor during driving [17]. Because this has become a major safety issue, the vast majority of research on inattention due to cell-phone use has concentrated on its implication on driving deficiencies.

However, the consequences of inattention are obviously not only specific to driving. For instance, cell phone users walk more slowly, change direction more frequently, and are less likely to acknowledge other people [18]. In fact, many people sense that using cellular phones and other electronic devices may have a strong cost on how we communicate and relate to each other. However, this cost has not been thoroughly studied empirically. The goal of this paper is to solve this empirical gap determining—in a quantitative manner—how human social interactions are affected by frequent interruptions based mostly on the use of electronic devices.

To this aim, we performed a two-player social game, with a large sample (N = 713 couples) playing simultaneously in two different experiments performed in theaters, during TEDx events. Each player of the dyad was assigned a different role. The speaker was asked to narrate a very engaging four-minutes story. Some listeners were given instructions to pay full attention to the speaker. Other listeners had to ignore the speaker mainly using his cell phone (text messages, Twitter, etc.). Finally, others listeners had to change their attitude from full attention to no attention for different time periods during the four-minute exercise. The speaker and the listeners only read the instructions of their role. However, the instructions of the listener included also a description of the speaker task. Thus, the listener is aware of all aspects of the experiment, while the speaker is not informed of the listener’s role. Following this treatment, we measured participant’s beliefs about the quality of the story and about the conversational partner.

## Methods

Experiments were performed on a large audience in two different theatres, in TEDx events. This experiment is part of an initiative referred to as TEDxperiments which aims to capitalize on TEDx events to construct knowledge on human communication. The first experiment was performed on September 27, 2013, in Buenos Aires, with an audience of 1200 people at TEDxRíodelaPlata (http://www.tedxriodelaplata.org). The second experiment was performed on October 9, 2013, in Rosario, with an audience of 900 people at TEDxRosario (http://www.tedxrosario.com.ar).

The theater research assistants handled the material (paper and pencil) to participate in the experiment. Participants were informed that participation in the experiment was completely voluntary and they could simply choose not to participate. Participants provided a verbal consent. They simply responded to the research assistants the willingness to participate. Due to the brevity of the experiment, participants did not sign a written consent form. Participants were explicitly assured that 1) they participation in the experiment was completely voluntary and that they could leave the experiment at any time and 2) that all the data was completely anonymous. Anonymity was assured since the questionnaires filled by participants did not have any personal information (name, age, gender) and were dropped in a common box. The procedures of the experiments described here were approved by the ethics committee of CEMIC (Centro de Educación Médica e Investigaciones Clínicas Norberto Quirno).

Each player was paired with the person sitting in the next row of the theater (to make it more probable that people would play with someone they did not know beforehand) and was given an envelope containing the instructions for two roles that were assigned randomly. Players were asked to open their role and then the game commenced. Videos of the game (see http://www.tedxriodelaplata.org/videos/tedxperiments) reflect a very strong commitment of players, in both theatres. Participants were assigned randomly to 6 different groups: 10% of the players were in the full attention group (the instruction was to pay attention all the time), another 10% were the none attention group (they were asked to ignore their conversational partner all the time). 80% of the participants were uniformly divided in four groups in which the listener ignored the speaker for a total of two minutes (half of the total story duration). The four groups were assigned different temporal patterns labeled [(++−−), (−−++), (+−+−), (−+−+)], where each symbol denotes a minute of the dialog and plus and minus signs index whether the listener attends the speaker (+) or not (−). After playing the game, players had one minute to fill an anonymous questionnaire which responding in range of 1 to 10 (1 is the minimum and 10 the maximum)

Speaker Questionnaire:

- About the story: whether they believed the story they told was entertaining (Question 1, Q1) and Emotive (Q2).
- About the conversational partner: whether their partner was an interesting (Q3), attractive (Q4) and enjoyable (Q5) person; whether the way they told the story was effective (Q6), recited fluidly and with good rhythm (Q7) and well perceived by the listener (Q8).

Listener Questionnaire:

- About the story: whether they believed the story they heard was entertaining (Question 1, Q1) and Emotive (Q2) and told fluidly (Q3).
- About the conversational partner: whether their partner was an interesting (Q4), attractive (Q5) and enjoyable (Q6) person.

The speaker and the listeners only read the instructions of their role. However, the instructions of the listener included also a description of the speaker task. Thus, the listener is aware of all aspects of the experiment (that the speaker is asked to tell a very important story and that they should ignore the speaker during specific times). Instead, the speaker is not informed of the listener’s role and has no way to know it. To assure that they did not guess that the listener was acting a role, we asked a random sample of participants (N = 170) whether they realized that the listener was acting a role. Only 3 participants (< 2%) responded positively.

Only pairs of players for whom we had all responses complete were considered for analyses, to assure that all comparisons could be paired. This left us with a total of 414 pairs in the first experiment and 299 in the second experiment.

## Results

After playing the game, the players completed a questionnaire responding several questions (on a 1–10 scale) about how they perceived their conversational partner and the story (see methods for a full description of the questionnaire).

**Is there a bias such that either speakers or listeners tend to judge the story and the partner differently?**

Listeners had a better opinion of the speaker than vice versa (average partner perception: speaker 6.68±0.08, listener 7.53±0.07). Similarly, the story (its quality, whether it was entertaining, recited fluidly) was better ranked by the receiver (average story perception: speaker 5.85±0.09, listener 6.67±0.09). These results were confirmed by a paired t-test comparing the scores within each speaker-listener pair which showed a highly significant difference (story: *t* = 9.08, *df* = 665, *p* < 1.18 × 10^−18^, Cohen’s *d* = 0.42, partner: *t* = 7.91, *df* = 615, *p* < 1.18 × 10^−14^, Cohen’s *d* = 0.41).

**Are speaker judgments determined by attention time?**

**Fig 1:**
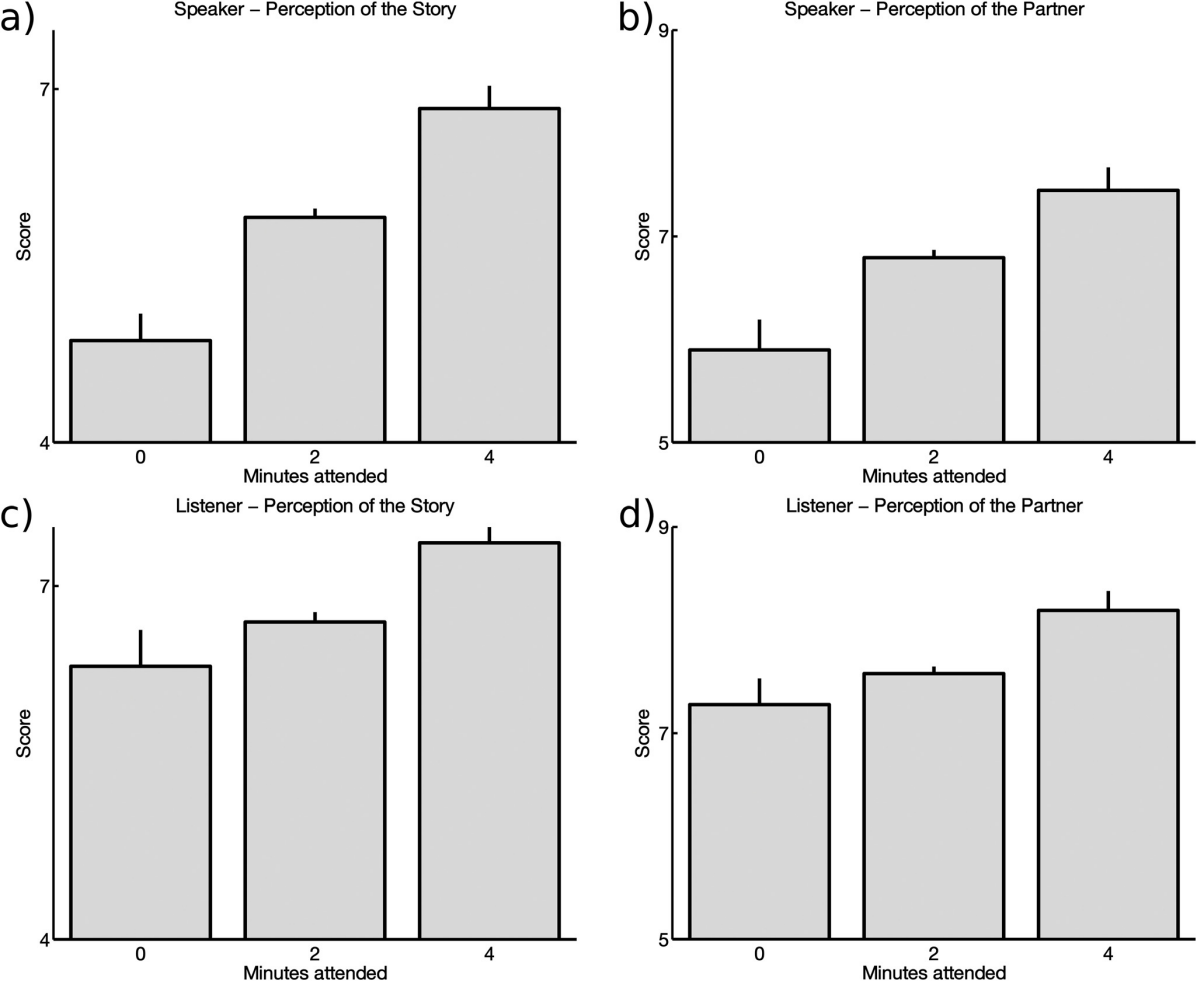
Speakers and Listeners judgments on quality of story and conversational partner increase as minutes of attention augment. a) The speaker’s judgments of the quality of the story. b) The speaker’s judgments of their partner. c) The listener’s judgments of the story. d) The listener’s opinion about the speaker.

The speaker’s judgments of the quality of the story they told and about their conversational partner increased markedly as the time of attention by the listener augmented (Fig 1, top panels a and b).

**Are listener judgments determined by attention time?**

Analogously, the listener’s judgments of the story they listened and their opinion about the speaker increased with the time of attention Fig 1, bottom panels c and d). This effect was more surprising since the listener was aware that ignoring the story was only due to task instructions and not because she considered the story or the conversational partner uninteresting. To confirm these results we submitted the data to two independent ANOVA, one for the story and one for the partner scores, with role (Speaker or Listener) and time of attention (0, 2 or 4 minutes as main factors). Both ANOVAS showed highly significant effects of the main factors (time and role) without interaction (Table 1). These results were replicated when analyzed for each of the two independent experiments (S1 Table)

**Table 1:** ANOVAs for the story and for the partner scores, with role (Speaker or Listener) and time of attention (0, 2 or 4 minutes as main factors).

	Story			Conversational Partner		
Factor	F	df	p	F	df	p
Role	16.47	2	**< 10** ^**-8**^	12.81	2	**< 10** ^**-8**^
Attended Time	26.53	1	**< 10** ^**-8**^	34.04	1	**< 10** ^**-8**^
Interaction	1.89	2	0.15	1.5	2	0.22

**Which judgments are affected by attention time?**

The questionnaire asked participants about different dimensions of the story and how it was told; including its flow and rhythm, whether it was entertaining and how emotional was its content. To investigate the effect of attended time in each question, we performed independent linear models (one for each question) with score as dependent variable and attended time as the main regressor. The sole score which did not increase monotonically with time of attention was the emotional content (Question 2, see Table 2).

**Table 2:** Linear regression of questions only about the story for speakers and listeners.

	Speaker					Listener		
Params	Q1	Q2	Q3	Q4	Q5	Q1	Q2	Q3
*α*	4.74	5.35	4.72	5.23	4.19	6.14	5.67	6.37
*β*	0.29	0.18	0.56	0.51	1.02	0.44	0.10	0.36
PP	**0.01**	0.16	**< 10** ^**-8**^	**< 10** ^**-8**^	**< 10** ^**-8**^	**< 10** ^**-8**^	0.45	**0.0041**

**Are judgment affected by whether attention is deployed at the beginning, at the end, or alternating throughout the story?**

The condition in which the listener ignored the speaker for two minutes (half of the total story duration) was organized in four different temporal patterns labeled [(++−−), (−−++), (+−+−), (−+−+)], where each symbol denotes a minute of the dialog and plus and minus signs index whether the listener pays attention to the speaker (+) or not (−). The aim of this factorial design is to investigate whether the dynamics of deployed attention (for fixed attended time) is pertinent for subjective constructs of the success of human communication. This experimental design controls in a factorial manner the durations of moments of attention and inattention (interval duration 1 or 2 minutes) and whether attention is deployed at the beginning and then fades out or conversely whether the listener first ignores the speaker and then attention grows (interval order: pay attention first or pay attention last).

We submitted the subjective scores of conversational partner and story perception to independent ANOVAs with order and duration and their interaction as independent factors. The data consistently showed that none of the subjective scores were sensitive to the effect of order or duration (S2 Table, none of the eight effects of interest reached significance or even reached marginal significance). Of course, it is impossible to discard a residual small effect size. However, the fact that this experiment is performed over a very large sample (*N* = 713 couples), and that the effect of attended time reached very high levels of significance, consistently in two independent experiments, suggests that overall the variance in subjective perception of human communication is largely determined by the total amount of attention and not on how it is distributed.

## Discussion

We investigated how neglect resulting from use of mobile devices affects human social perception. Our results show that speakers grade the quality of the story and the sympathy towards the listener in proportion to the time they are paid attention. The quantitative measure of this results points out that 1) this is a highly significant effect (perception of the story by a speaker changes from ≈ 4.86/10 to ≈ 6.83/10 from no to full attention) which is consistent across experiments and reaches very high levels of significance and 2) the effect is mostly independent on whether inattention alternates in time or is collapsed in long lasting episodes.

More surprisingly, the listener qualification of the story and the sympathy they report towards the speaker also varied with the amount of attended time. This result was less expected to us prior to the experiment because the listener is fully aware of the fact inattention solely responds to task instructions and not to the merits of the story. The current data cannot fully narrow the principles leading to this observation, but there are two parsimonious (and not mutually exclusive) explanations. First, this observation naturally results as a way to avoid cognitive dissonance [19]. Ignoring and praising someone at the same time (even if ignorance is presumably not related to the merits of the speaker) yields to two contradictory beliefs and values, which is known to be a source of cognitive stress and discomfort that tends to be avoided implicitly [19]. A similar interpretation of this finding is that it may be affected by demand effects, usually defined in economic experiments to refer to changes in behavior by experimental subjects due to cues about what constitutes appropriate [20]: the listener may think that if the experimenter asks him/her to ignore the speaker, a response of less interest is expected.

Second, it is possible that upon being ignored, the speaker changes his attitude, gives up on the story and makes it worse in which case the receiver’s scores would reflect a genuine deterioration of the quality of the story (and probably of the way the speaker looks) as they progressively remove attention.

Studies examining children raised in severe cognitive neglect have consistently shown marked cognitive development deficits related to major decreases in individual attention and emotional affection [21]. Our study can be seen as a way to address the effect of inattention in subjective beliefs in a much more frequent and less extreme condition: a person being partially ignored while telling a very meaningful story.

One consequence of inattention and distraction during human communication is the disruption of ostension. Ostensive signals, which include among others directing gaze, raising the eye-brows and changing the tone of voice, constitute a natural protocol to convey pertinence in human communication [22]. In natural human communication, ostensive signals index the reliability and trustability of the communicator as well as the pertinence of the communicated message [22]. Hence, a natural prediction of the theory is that a person ignored while telling a story would generate negative beliefs about the recipient (the person breaking ostension) and also about the story being told. Our work confirms this hypothesis both from the point of view of the emitter and the receiver of the communicated message, quantifying the cost of inattention.

Finally, our analysis showed that the temporal distribution of attention has a negligible effect on the communicators’ beliefs. Different theories predicted opposing outcomes from this temporal manipulation of attention.

One principle is that communication begins with a handshaking protocol, a statement by which the agents agree upon their intention to communicate [23]. From this principle it derives that inattention in the first moment of the dialog should lead to a worse perception of the receiver (the lack of politeness of sustaining a hand-shaking protocol). Instead, inattention during the last moments of the dialogs does not break the protocol of communication and may be instead attributed by the emitter to the fact that the story was uninteresting. A dancing metaphor may help anchoring this idea. If a person invites another to dance and the invitee (the recipient) rejects the invitation, the emitter (the person making the invitation) may make the inference that the invitee was not polite. Instead, if the receiver accepts the invitation but breaks the dance by the end of it, the natural inference is that the dance was not good enough to sustain attention and hence blame is on the message (the dance, or the story) and not on the conversational partner.

Alternatively, it may be reasoned that—as observed in other domains of cognition—retrospective beliefs are dominated by the perception at the last episodes [24]. For instance, Kahneman (1993) found that subjects retrospectively prefer a treatment in which a fixed amount of pains is followed by a lower dose than when it is followed by no pain. This implies that participants do not accumulate the total amount of discomfort but instead generate beliefs based on fragments of the experience, largely dominated by the last episode. In our experiment, if the data were dominated by this principle we would expect that participants’ beliefs would be affected by the temporal distribution of attention (experience would be ranked worse when the listener neglects the speaker during the end of the story) and less so by the total amount of attention. Instead, our data show that the distribution of attended time plays a negligible role in the variance of subjective perception of the story and of the conversational partner.

In summary, a quantitative analysis following a face to face brief (4 minutes) communication shows that the total amount of attention is the major factor driving subjective beliefs about the message (the story being told) and of the conversational partner. The effect is observed on both the emitter and the receiver and is mostly independent on how attention is distributed in time. Interruptions during day to day communication between peers and also with children are extremely frequent. Our data should provide a note of caution, by signaling the consequences of these windows of neglect on the teller and receiver of a story.

## Supporting Information

S1 Table Story and the partner scores. ANOVAs for the story and for the partner scores at each experiment location (Buenos Aires and Rosario), with role (Speaker or Listener) and time of attention (0, 2 or 4 minutes as main factors).
(PDF)

S2 Table Subjective perception of the story and partner. ANOVAs for the subjective perception of story and partner for each role, with order (whether listener finished paying attention or not) and temporal pattern of attentions (2 interleaved blocks of 2 minutes or 4 interleaved blocks of 1 minute).
(PDF)
