# Supplementary material for: Neglect in Human Communication: Quantifying the Cost of Cell-Phone Interruptions in Face to Face Dialogs
Source: PLoS One. 2015 Jun 3;10(6):e0125772. doi: 10.1371/journal.pone.0125772 (PMC4454692; doi:10.1371/journal.pone.0125772)
Supplement: S1 Table — ANOVAs for the story and for the partner scores at each experiment location (Buenos Aires and Rosario), with role (Speaker or Listener) and time of attention (0, 2 or 4 minutes as main factors). (PDF) [file pone.0125772.s001.pdf]

Neglect in human communication: quantifying the cost of cell-phone interruptions in face to face dialogs (Supplemental material)

Lopez-Rosenfeld, Matías[1], Calero, Cecilia[2], Fernandez Slezak, Diego[1], Garbulsky, Gerry[3], Bergman, Mariano[2], Trevisan, Marcos[4], and Sigman, Mariano[2]

[1]Laboratorio de Inteligencia Artificial Aplicada, Departamento de Computación, Facultad de Ciencias Exactas y Naturales, Universidad de Buenos Aires, Buenos Aires, Argentina
[2]Universidad Torcuato Di Tella, Buenos Aires, Argentina
[3]El Mundo de las Ideas, Buenos Aires, Argentina
[4]Departamento de Fisica, Facultad de Ciencias Exactas y Naturales, Universidad de Buenos Aires, Buenos Aires, Argentina

Supporting Information

Supplementary Table 1. ANOVAs for the story and for the partner scores at each experiment location (Buenos Aires and Rosario), with role (Speaker or Listener) and time of attention (0, 2 or 4 minutes as main factors).

Buenos Aires						
	Story			Conversational Partner		
Factor	F	df	p	F	df	p
Role	11.28	2	$\mathbf{< 10^{-8}}$	6.25	1	**0.002**
Attended Time	14.64	1	**0.0001**	10.4	1	**0.0013**
Interaction	0.72	2	0.49	0.45	1	0.64
**Rosario**						
	Story			Conversational Partner		
Factor	F	df	p	F	df	p
Role	4.82	1	**0.0084**	8.47	1	**0.0002**
Attended Time	12.08	1	**0.0005**	27.69	1	$\mathbf{< 10^{-8}}$
Interaction	1.47	1	0.23	3.44	1	0.03
