# Supplementary material for: Neglect in Human Communication: Quantifying the Cost of Cell-Phone Interruptions in Face to Face Dialogs
Source: PLoS One. 2015 Jun 3;10(6):e0125772. doi: 10.1371/journal.pone.0125772 (PMC4454692; doi:10.1371/journal.pone.0125772)
Supplement: S2 Table — ANOVAs for the subjective perception of story and partner for each role, with order (whether listener finished paying attention or not) and temporal pattern of attentions (2 interleaved blocks of 2 minutes or 4 interleaved blocks of 1 minute). (PDF) [file pone.0125772.s002.pdf]

Neglect in human communication: quantifying the cost of cell-phone interruptions in face to face dialogs (Supplemental material)

Lopez-Rosenfeld, Matías[1], Calero, Cecilia[2], Fernandez Slezak, Diego[1], Garbulsky, Gerry[3], Bergman, Mariano[2], Trevisan, Marcos[4], and Sigman, Mariano[2]

[1]Laboratorio de Inteligencia Artificial Aplicada, Departamento de Computación, Facultad de Ciencias Exactas y Naturales, Universidad de Buenos Aires, Buenos Aires, Argentina
[2]Universidad Torcuato Di Tella, Buenos Aires, Argentina
[3]El Mundo de las Ideas, Buenos Aires, Argentina
[4]Departamento de Fisica, Facultad de Ciencias Exactas y Naturales, Universidad de Buenos Aires, Buenos Aires, Argentina

Supporting Information

Supplementary Table 2. ANOVAs for the subjective perception of story and partner for each role, with order (whether listener finished paying attention or not) and temporal pattern of attentions (2 interleaved blocks of 2 minutes or 4 interleaved blocks of 1 minute).

Speaker						
	Story			Conversational Partner		
Factor	F	df	p	F	df	p
Order	1.54	1	0.21	0.2	1	0.66
Pattern	3.14	1	0.07	0.76	1	0.38
Interaction	0.28	1	0.6	2.87	1	0.09
Listener						
	Story			Conversational Partner		
Factor	F	df	p	F	df	p
Order	2.92	1	0.09	5.89	1	**0.01**
Pattern	4.25	1	**0.04**	2.92	1	0.09
Interaction	0.14	1	0.71	0.11	1	0.74
